# Dietary salt and protein intake and urinary cystine excretion in patients with cystinuria

**DOI:** 10.1093/ckj/sfaf329

**Published:** 2025-10-29

**Authors:** Francesca Bermond, Laura Fabbrini, Laura Rivoli, Andrea Spasiano, Marta Leporati, Michele Petrarulo, Andrea Ricotti, Lucia Borsotti, Martino Marangella, Domenico Cosseddu, Pietro Manuel Ferraro, Corrado Vitale

**Affiliations:** Nephrology and Dialysis Unit, Medical Department, A.O. Ordine Mauriziano di Torino, Turin, Italy; Nephrology and Dialysis Unit, Medical Department, A.O. Ordine Mauriziano di Torino, Turin, Italy; Nephrology and Dialysis Unit, Medical Department, A.O. Ordine Mauriziano di Torino, Turin, Italy; Section of Nephrology, Department of Medicine, Università degli Studi di Verona; Nephrology Unit, Azienda Ospedaliera Universitaria Integrata Verona, Verona, Italy; Kidney Stone Laboratory, A.O. Ordine Mauriziano di Torino, Turin, Italy; Kidney Stone Laboratory, A.O. Ordine Mauriziano di Torino, Turin, Italy; Clinical Trial Unit, A.O. Ordine Mauriziano di Torino, Turin, Italy; Clinical Trial Unit, A.O. Ordine Mauriziano di Torino, Turin, Italy; Fondazione Scientifica Mauriziana O.N.L.U.S., Turin, Italy; Kidney Stone Laboratory, A.O. Ordine Mauriziano di Torino, Turin, Italy; Section of Nephrology, Department of Medicine, Università degli Studi di Verona; Nephrology Unit, Azienda Ospedaliera Universitaria Integrata Verona, Verona, Italy; Nephrology and Dialysis Unit, Medical Department, A.O. Ordine Mauriziano di Torino, Turin, Italy

**Keywords:** cystinuria, dietary proteins, kidney stone disease, nephrolithiasis, sodium intake

## Abstract

**Background:**

Cystinuria is a rare autosomal recessive disorder characterized by impaired renal reabsorption of cystine and dibasic amino acids, leading to recurrent nephrolithiasis. While dietary salt and protein restriction are commonly recommended, evidence supporting their effectiveness in reducing urinary cystine excretion is limited. This study investigated whether intra-individual changes in dietary salt and protein intake are associated with changes in cystine excretion over time.

**Methods:**

We conducted a retrospective cohort study of 41 adult patients with recurrent cystine stones treated at a tertiary kidney stone clinic between 2004 and 2023. All patients underwent five 24-hour urine collections at intervals of 6–12 months. Urinary sodium and urea excretions were used as surrogates for salt and protein intake, respectively. Mixed-effects linear regression models assessed within-person associations between dietary intake and urinary cystine excretion, adjusting for age, sex, and time.

**Results:**

Cystine excretion showed considerable intra-individual variability (intraclass correlation coefficient: 0.457). An increase in urinary urea of 3.5 g/24 h (reflecting ∼10 g/day higher protein intake) was associated with a 164 µmol/24 h increase in cystine excretion (95% CI: 57 to 271, *P* = .003). In contrast, a 17 mmol/24 h increase in urinary sodium (∼1 g/day salt intake) was associated with a non-significant 46 µmol/24 h increase in cystine excretion (95% CI: −5 to 97, *P* = .081).

**Conclusions:**

Protein intake is moderately associated with urinary cystine excretion, whereas salt intake has minimal effect. These findings suggest that, while protein moderation may contribute to cystine reduction, fluid intake and urine alkalinization remain the primary determinants of urinary cystine levels.

KEY LEARNING POINTS **What was known:**

- Cystine excretion is influenced by fluid intake, urine pH, and possibly by dietary protein and salt, but longitudinal data within individuals with cystinuria were lacking.

**This study adds:**

- Protein intake has a moderate effect on urinary cystine excretion, whereas salt intake has minimal effect.
- However, protein explains only part of the intra-individual variability, highlighting the predominant roles of fluid intake and urine alkalinization.

**Potential impact:**

- These findings may help refine dietary recommendations and guide future interventions, indicating that reducing protein intake can moderately decrease cystine excretion, while focusing on more impactful factors such as fluid intake and urine alkalinization.

## INTRODUCTION

Cystinuria is caused by pathogenic mutations in the genes that encode a common transporter made up of two subunits (rBAT, encoded by the *SLC3A1* gene, and b0,+AT, encoded by the *SLC7A9* gene), which leads to impaired cystine and dibasic amino acid reabsorption in the kidney tubules. The estimated global prevalence of this condition is ∼1 in 7000 newborns [1].

In normal individuals, daily urinary cystine excretion is <200 µmol. Since the upper limit of cystine solubility in urine is ∼1000 µmol/l at pH ≥ 7.0, normal subjects do not form cystine stones [2, 3].

Patients with biallelic mutations (genotypes AA and BB) experience recurrent kidney stone disease, as a consequence of urinary cystine excretion typically exceeds 1600 µmol/24 h, usually ranging from 2500 to >6000 µmol/24 h [4].

For patients with cystinuria, preventing kidney stone formation involves lowering the concentration of cystine in the urine and increasing its solubility. This can be achieved by modifying both diet and fluid intake, alkalinizing urine and, if necessary, using drugs containing thiols such as tiopronin or penicillamine [3, 4].

To lower urinary cystine concentration and improve its solubility, it was recommended to increase urinary volume to >3 l per day and to raise urine pH between 7.5 and 8.0 through alkali supplementation [5].

It has also been hypothesized that a low dietary intake of animal protein may be beneficial, as they represent the primary nutritional source of cysteine and its metabolic precursor, methionine [6]. This approach could help reduce both cystine excretion and the acidifying effect on urine caused by the metabolism of sulfur-containing amino acids [7, 8].

In particular, Goldfarb *et al.* identified a significant correlation between urea and cystine excretions in a dataset comprising 79 urine samples. However, the study had a cross-sectional design and did not incorporate repeated urine measurements over time [9].

Similarly, Rodman *et al.* quantified the effects of protein on urine cystine excretion by switching seven adult patients from a low-protein diet, averaging 54 g of protein per day, to a high-protein diet, of at least 140 g of protein per day. These modifications led to a notable increase in mean cystine excretion, rising from 4890 to 6130 µmol/24 h [7]. Specifically, each 10 g/day increment in mixed protein intake was associated with a 144 µmol/day increase in cystine excretion, which constituted ∼3% of the cystine excretion observed under a low-protein diet.

It has also been suggested that reducing sodium intake to below 100 mEq/day may contribute to decreasing cystine excretion [3, 10–12]. Specifically, Goldfarb *et al.* identified a significant correlation between sodium and cystine excretions in the aforementioned study, although the previously reported limitations make the study not suitable for investigating within-person correlations in sodium and cystine excretions [9]. Additionally, a small study conducted by Jaeger *et al.* demonstrated that reducing the dietary sodium intake from 300 to 150 mEq/day led to a significant decrease in cystine excretion, ∼650 µmol/day. That is, a 17 mEq reduction in sodium intake (corresponding to a 1 g reduction in salt intake [13]) was associated with a 74 µmol decrease in cystine excretion [10]. Norman *et al.* also documented that a reduction in sodium intake of 100 mEq/day corresponded to a decrease in urinary cystine levels of ∼1200 µmol/day. Specifically, this indicates that for every 1 g reduction in salt intake, there was a subsequent decrease of 204 µmol in cystine excretion [14].

 Furthermore, in a study by Lindell *et al.*, 13 patients were evaluated both at baseline and during three distinct periods of a sodium-controlled diet. It was found that for each 1-mEq increase in sodium excretion, the average cystine excretion increased by 3.1 µmol. This implies that an increase of 1 g in salt intake could lead to a 52.7 µmol increase in cystine excretion [15].

However, no clinical trials have definitively demonstrated that dietary modifications effectively lower cystine excretion or prevent stone formation.

Additionally, in healthy individuals, changes in dietary salt intake did not affect cystine excretion. Jaeger *et al.* did not observe significant alterations in cystine excretion among five healthy subjects when sodium intake was increased from 30 to 400 mEq/day [10]. More recently, Siener *et al.* found no correlation between dietary sodium intake and urinary cystine excretion in a study involving 10 healthy men, observed across three different standardized diets [16].

This study aimed to conduct a retrospective analysis of urinary cystine excretion variability over time in patients with recurrent cystine nephrolithiasis. It also sought to investigate whether changes in dietary intake of salt and protein could explain the observed variations in cystine excretion.

## MATERIALS AND METHODS

This retrospective cohort study examined adult patients with recurrent cystine nephrolithiasis referred to the Kidney Stone Clinic at the “Azienda Ospedaliera Ordine Mauriziano di Torino” (Turin, Italy) between 1 January 2004 and 31 December 2023.

The study received approval from the institution’s research ethics board (“Comitato Etico Territoriale Interaziendale AOU Città della Salute e della Scienza di Torino,” number 00089/2025, 14 April 2025); all participants provided informed consent.

Patients were selected based on the following criteria: (i) any cystine in at least one stone, either passed spontaneously or surgically removed, through Fourier-transform infrared spectroscopy; (ii) simultaneous measurements of urinary creatinine, cystine, sodium, and urea on five consecutive metabolic assessments carried out every 6–12 months; (iii) age between 18 and 80 years during the observation period; (iv) creatinine clearance of at least 45 ml/min throughout the observation period; and (v) in patients undergoing therapy with thiols (tiopronin, penicillamine) the daily dosage must have been unchanged throughout the observation period.

All participants were instructed to maintain a daily water intake of 3–4 l and adhere to a dietary regimen rich in fruits and vegetables (at least one serving at each meal), with a balanced intake of animal and vegetable protein. The consumption of salty food was discouraged. For each patient, the results from five consecutive assessments of 24-hour urinary metabolic profile were retrieved from our database.

Urine collections were performed at home, by dividing each micturition into two plastic bottles. The first bottle was pre-filled with 7 ml of 37% hydrochloric acid as a preservative, and urine was analyzed for sodium (ion-selective electrode), urea (routine method) and creatinine (enzymatic method). The second bottle was pre-filled with 2 ml of 20% chlorhexidine as an antibacterial preservative, and urine was assayed for cystine (reverse phase liquid chromatography coupled with a fluorescence detector) and pH (direct potentiometry). Creatinine clearance was calculated from plasma and urine concentrations of creatinine and urine volume according to the standard formula. Dietary salt intake was inferred from urinary sodium excretion, with a 17 mmol/24 h increase in urinary sodium indicating an increase in salt consumption of 1 g/day [13]. A 3.5 g/24 h increase in urinary excretion of urea was considered indicative of an increase in protein intake of ∼10 g/day [17].

### Cystine assay

Urinary cystine was quantitated by the commercial KIT ClinRep^®^ (BSN; RECIPE Chemicals; München, Germany). Both cysteine and mixed disulfides are reduced into sulfur-containing monomers (R-SH), which undergo derivatization with a halogenobenzofurazan, resulting in fluorescent compounds detectable by reverse phase liquid chromatography coupled with a fluorescence detector. Our assay measures total cystine and does not differentiate between free cystine and cysteine bound to thiol drugs such as tiopronin. Briefly, the method employs a strong reducing agent to break all disulfide bonds, including those between cysteine molecules, between cysteine and tiopronin, and between tiopronin molecules. After reduction, the resulting free cysteine is quantified, and total cystine is calculated as half of the measured cysteine monomer concentration. This provides a reliable measure of total cystine suitable for the objectives of our study.

The analytical steps are summarized as follows: 50 µl of the sample were fivefold diluted with ultrapure water and spiked with 25 µl of homocysteine solution (250 µmol/l, internal standard). Therefore, 12.5 µl of the reducing reagent and 50 µl of ultrapure water were added. The samples were centrifuged, and 25 µl of surfactant were transferred into a micro-vial. Fifty microliters of buffer reagent and 25 µl of derivatizing reagent were added. Samples were centrifuged and incubated for 60 min at 60°C. Subsequently, the samples were cooled for 5 min at 2–8°C and injected in the HPLC system.

The instrumental analysis was performed using an Agilent Technologies (Santa Clara, CA, USA) HPLC system, model 1200, coupled with a fluorescence detector. The system was controlled using the ChemStation software (Agilent Technologies, Santa Clara, CA, USA). The chromatographic separation was performed using the column and mobile phase of the BSN Recipe kit. The flow rate was 1 ml/min and the injection volume was 20 µl. The fluorescence signals were measured with excitation at 385 nm and emission at 515 nm.

### Statistical analysis

Continuous variables were reported as means with standard deviations (SDs) or medians with 25th and 75th percentiles; categorical variables as frequencies and percentages.

To analyze the association between changes in intakes of salt and protein (proxied by 24 h urinary excretions of sodium and urea, respectively) and urinary excretion of cystine within the same individual, we used mixed linear regression models with random intercept and time slopes for each patient.

Models were adjusted for age, sex, and visit (modeled as a categorical variable); between and within-person effects of salt and protein intake on cystine excretion were partitioned, and coefficients for the within-person effects were reported together with their 95% confidence interval (CI). Those can be interpreted as the change in urine cystine excretion associated with a given change over time in either urine sodium or urea within the same person.

Non-linearity was checked by comparing the full model with linear terms for urine sodium and urea to a model with cubic splines for urine sodium and urea. Effect modification was explored by including an interaction term between urine sodium and urea.

To estimate between and within-person variance of urine cystine and to partition sources of variance, we computed the intraclass correlation coefficient (ICC) for the unconditional model (the model that did not include any covariate), the covariate-only model (the model including age, sex and visit) and the full model (the model including also urine sodium and urea).

A two-tailed P value <.05 was considered statistically significant. All analyses were performed with R version 4.4.2.

## RESULTS

Baseline characteristics of the patients are reported in Table 1. The study included 41 patients [27 (65.9%) men], with a mean (SD) age of 49 (16) years at the beginning of the observation period.

**Table 1:** Characteristics, therapies, and urine excretions of enrolled patients.

	Overall (*N* = 41)
Sex	
F	14 (34.1%)
M	27 (65.9%)
Age (years)	
Mean (SD)	48.8 (15.5)
Median [min, max]	53.0 [20.0, 78.0]
Therapy	
No	5 (12.2%)
Penicillamine	3 (7.3%)
Tiopronin	33 (80.5%)
Urine cystine excretion (µmol/24 h)	
Mean (SD)	3560 (1180)
Median [min, max]	3440 [1570, 6390]
Urine sodium excretion (mmol/24 h)	
Mean (SD)	174 (72.8)
Median [min, max]	183 [27.0, 370]
Urine urea excretion (g/24 h)	
Mean (SD)	21.0 (8.03)
Median [min, max]	19.1 [7.90, 51.4]
Missing	4 (9.8%)

**Figure 1:**
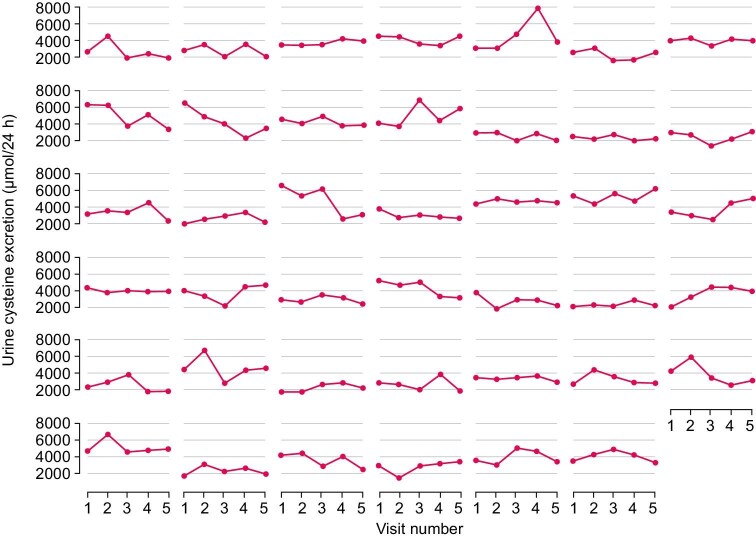
Trellis plot of urine cystine excretion.

All patients took potassium citrate as urinary alkalinizer, in daily doses ranging from 2 to 6 g. Thirty-three patients received tiopronin (daily dose ranging between 1000 and 2000 mg), while two patients received penicillamine (daily dose ranging from 900 to 1500 mg). Individual values of urine cystine excretion, collected over a total of 2169 patient-months [average (SD) 53 [10] patient-months], are reported in Fig. 1.

After comparing models with both random intercept and time slopes with models including only random intercepts, we found that the former did not fit the data significantly better than the latter, thus used random intercept models for all the analyses.

Results of the regression models are reported in Table 2. After full adjustment, within-person changes in urine sodium were not associated with significant changes in urine cystine excretion: a 17 mmol/24 h increase in urine sodium (corresponding to an increase in salt intake of ∼1 g/day [13]) was associated with an increase in urine cystine of only 46 µmol/24 h (95% CI −5, 97 µmol/24 h; *P* = .081); on the other hand, changes in urine urea were directly associated with urine cystine excretion: a 3.5 g/24 h increase in urine urea (corresponding to an increase in protein intake of about 10 g/day) was associated with an increase in urine cystine of 164 µmol/24 h (95% CI 57, 271 µmol/24 h; *P* = .003).

**Table 2:** Effect of a 17 mmol/24 h change in urine sodium and 3.5 g/24 h change in urine urea on urine cystine excretion and cystine SS.

	Change in urine cystine (µmol/24 h)	Change in SS cystine (%)
Change in urine sodium	46 (−5, 97) *P* = .081	−0.5 (−2.5, 1.6) *P* = .665
Change in urine urea	164 (57, 271) *P* = .003	4.9 (0.5, 9.5) *P* = .031

We found no significant interaction between urine sodium and urea on urine excretion of cystine (*P* value for interaction .76). Spline analysis showed that non-linear associations could be excluded (*P* value for the comparison of linear vs spline models .10).

The urinary excretion of cystine varied considerably within the same individual over time, with an ICC for the unconditional model (the model that did not include any covariate) of 0.457; in other words, 54.3% of the total variance of cystine could be explained by within-person factors. Addition of urine sodium and urea to the covariate-only model additionally explained 6.3% of the total variance and 17.1% of the within-person variance.

## DISCUSSION

Our findings suggest that dietary protein intake has a moderate effect on cystine excretion. In contrast to earlier studies, the influence of sodium intake was minimal and not statistically significant.

In contrast to the aforementioned study by Rodman *et al.*, our research examined the effect of protein by considering the urinary urea excretion rather than the diet composition. Our results, however, were remarkably similar, as an increase in protein intake of ∼10 g/day resulted in a rise in urinary cystine of 164 µmol/24 h, which accounts for ∼5% of the average cystine excretion in the study population.

When the urine pH is 7 or higher, the solubility threshold for cystine is ∼1000 µmol/l. Beyond this threshold, an increase in cystine excretion of ∼170 µmol could be offset by a corresponding increase in urinary volume of <200 ml. Then we concluded that the clinical impact of protein intake is ultimately low to moderate.

With regard to salt intake, in our analysis, conducted on a larger cohort of patients compared to previous studies, it was observed that a daily increase of 17 mEq in urinary sodium was associated with just a 46 µmol/24 h increase in urinary cystine. Although this finding aligns with the results of both Jaeger *et al.* [10] and Lindell *et al.* [15], it did not achieve statistical significance (*P* = .081).

Consequently, the anticipated correlation between sodium intake and cystine excretion in individuals with cystinuria was not confirmed. This relationship remains challenging to elucidate, as the tubular transport of cystine operates independently of sodium transport [18]. Interestingly, our analysis simultaneously adjusted for protein intake, suggesting that the earlier findings of a significant association between salt intake and cystine excretion might have been confounded by protein intake, which is usually directly related to salt intake.

In contrast, the findings of our study are consistent with prior research concerning cystine excretion in healthy individuals. Overall, the observed correlation between urea and cystine excretions substantiates the recommendation regarding appropriate protein intake for patients with cystinuria [4, 11, 12].

Conversely, the minimal effects, if any, of sodium excretion do not corroborate the purported benefits of salt restriction in the clinical management of cystinuria. Of course, moderate salt intake remains a key element to prevent complications such as high blood pressure and chronic kidney disease, both conditions common in patients with cystinuria [19].

In summary, our findings indicate that dietary protein restriction may hold less significance in the conservative management of cystinuria when compared to the importance of substantial fluid intake and alkali supplementation.

A significant portion of the intra-individual variability in urinary cystine excretion remains unexplained. Several factors deserve consideration and may provide valuable insights for future research.

Our study has several strengths, including the large sample size for a rare condition, the systematic and accurate determination of urine chemistries over a long time span which allowed us to estimate within-subject effects, the elimination of confounding effects by thiols. It also has limitations, including the observational nature and the lack of direct dietary information.

However, substantial gaps remain in understanding the dietary management of cystinuria. To date, no large prospective studies have evaluated the effects of specific diets, such as fixed animal protein, vegetarian, or low-salt diet, on urinary cystine excretion. Addressing this knowledge gap would be highly valuable, as patients frequently inquire about specific dietary strategies. Future studies should incorporate longitudinal monitoring of relevant urinary biomarkers, including cystine, sodium, and urea, to provide mechanistic insights and generate robust, evidence-based dietary recommendations for individualized patient management.

In conclusion, in our study of 41 patients with cystinuria, protein intake is moderately associated with urinary excretion of cystine, whereas salt intake is not significantly associated with cystine excretion.

## Data Availability

The data were collected using REDCap^®^ (Research Electronic Data Capture), a secure web application for building and managing online surveys and databases. All data generated or analyzed during this study are included in this published article; further inquiries can be directed to the corresponding author.
